# Low fasting plasma glucose level as a predictor of new-onset diabetes mellitus on a large cohort from a Japanese general population

**DOI:** 10.1038/s41598-018-31744-4

**Published:** 2018-09-17

**Authors:** Emi Ogata, Koichi Asahi, Satoshi Yamaguchi, Kunitoshi Iseki, Hiroaki Sato, Toshiki Moriyama, Kunihiro Yamagata, Kazuhiko Tsuruya, Shouichi Fujimoto, Ichiei Narita, Tsuneo Konta, Masahide Kondo, Yugo Shibagaki, Masato Kasahara, Tsuyoshi Watanabe, Michio Shimabukuro

**Affiliations:** 1 Department of Diabetes, Endocrinology and Metabolism, School of Medicine, Fukushima Medical University, Fukushima, Japan; 2 Steering Committee of Research on Design of the Comprehensive Health Care System for Chronic Kidney Disease (CKD) Based on the Individual Risk Assessment by Specific Health Check, Fukushima, Japan; 3 Department of Cardiology, Tomishiro Central Hospital, Okinawa, Japan

## Abstract

Although fasting plasma glucose levels <70 mg/dL are associated with a high incidence of cardiovascular disease (CVD), whether there is any risk of new-onset diabetes mellitus owing to fasting plasma glucose at this range has not been clarified. We measured the odds ratio (OR) of new-onset diabetes mellitus relative to fasting plasma glucose levels at various ranges in a nation-wide Japanese population with and without CVD history. Of 186,749 participants without diabetes in 2008, 171,408 had no history of CVD, while 15,341 did. Participants were classified into 8 categories according to their fasting plasma glucose levels. Unadjusted and multivariable-adjusted logistic regression models were used to measure the OR of new-onset diabetes mellitus in the 3-year follow up. In all participants, multivariable-adjusted OR increased when fasting plasma glucose levels were <70 mg/dL or 90–125 mg/dL. Participants without CVD showed increased OR when glucose levels were <70 mg/dL or 90–125 mg/dL. Participants with a history of CVD showed increased OR with glucose levels of 95–125 mg/dL. The risk of new-onset diabetes mellitus is higher when fasting glucose levels are <70 mg/dL, indicating that the paradox of fasting glucose seeks a new risk stratification for new-onset diabetes mellitus.

## Introduction

The risk of new-onset diabetes mellitus increases as fasting plasma glucose levels increase within normoglycaemic levels between 80 mg/dL and 125 mg/dL^1–3^. However, the incidence of diabetes mellitus in those with fasting plasma glucose levels lower than 80 mg/dL has not been clarified.

Subjects with fasting plasma glucose levels below 70 mg/dL have a high susceptibility to cardiovascular diseases (CVD)^4–6^. Wei *et al*. conducted two prospective studies and showed the risk of cardiovascular disease mortality increased in participants with low fasting plasma glucose^4^. The risks for the various CVD outcomes increased with fasting plasma glucose levels below 70 mg/dL and above 110 mg/dL in the Korean cancer prevention study (KCPS) which is a prospective cohort study in Korea^6^. The correlation between fasting plasma glucose levels and CVD risk shows a J shape curve^5,6^. The underlying mechanism(s) of the CVD risk in low fasting glucose levels is not clear^7^. Wei *et al*. hypothesized that long-term exposure to low fasting plasma glucose levels may serve as a risk factor for CVD mortality, due to the resulting abnormal cardiac activity and thrombosis, particularly in patients with atherosclerosis^4^. Tanne *et al*. suggested that hypoglycaemia or rapid changes in plasma glucose may lead to elevated counter-regulatory hormone levels, such as adrenaline and noradrenaline, which induce vasoconstriction and platelet aggregation^8^. The changes observed at low fasting plasma glucose levels (<70 mg/dL) may also impair glucose metabolism, but this notion has not yet been evaluated. Clinically, spontaneous hypoglycaemia is considered to be caused by several clinical conditions such as insulinomas, non-insulinoma pancreatogenous hypoglycemia syndrome, insulin autoimmune syndrome, postprandial hypoglycemia (reactive hypoglycemia), primary adrenal insufficiency, hypopituitarism, and critical illness^9^. Spontaneous hypoglycaemia, in either way, symptomatic or asymptomatic, could also occur as an early manifestation of diabetes mellitus^10^. However, the relationship between spontaneous hypoglycaemia and the incidence of new-onset diabetes mellitus has not been fully clarified.

The purpose of this study was to examine whether the incidence of new-onset diabetes mellitus increases when fasting plasma glucose levels are below 70 mg/dL, and if so, whether this may be correlated to CVD history.

## Results

### General characteristics

Among all participants (n = 303,654) in 2008 (Dataset1, Supplementary Fig. 1), ones who visited only in 2008 (n = 78,334) or showed logical outliers for fasting plasma glucose (n = 536) were excluded (Dataset 2) and the main analysis were done on the complete case (Dataset 3, n = 186,749) excluded for missing data (n = 38,035). The characteristics of total participants (Dataset 3) are shown in Table 1. The average age was 63.6 years; 39.1% of participants were men, and the average BMI was 23.0 kg/m^2^. BMI, waist circumference, SBP, DBP, LDL-C, hypertension and walking >1 hour/day were linearly increased across all groups. Meanwhile, age, male gender, triglycerides, ALT, γGTP, dyslipidaemia and everyday drinking were linearly increased and HDL-C was linearly decreased, except for those with fasting plasma glucose levels <70 mg/dL. Current smoking was increased in the category with fasting glucose levels <70 and ≥90 mg/dL.

**Table 1:** Baseline characteristics of total subjects.

Fasting plasma glucose	Total	ANOVA	<70 mg/dL	70–79 mg/dL	80–84 mg/dL	85–89 mg/dL	90–94 mg/dL	95–99 mg/dL	100–109 mg/dL	110–125 mg/dL
**Total subjects (n = 186,749)**										
n	186,749		690	9,123	20,718	37,079	41,744	33,078	32,253	12,064
Age, years	63.6 (7.9)	<0.001	62.9 (8.4)	62.2 (8.8)^‡^	62.7 (8.5)^‡^	63.2 (8.2)	63.6 (7.8)^‡^	63.9 (7.6)^‡^	64.2 (7.3)^‡^	64.8 (6.8)^‡^
% Male	39.1%		32.5%	26.1%^‡^	27.7%^‡^	32.0%	37.3%^‡^	43.2%^‡^	50%^‡^	57.2%^‡^
BMI, kg/m^2^	23.0 (3.1)	<0.001	21.9 (3.1)^‡^	21.8 (3.0)^‡^	22.2 (3.0)^‡^	22.6 (3.0)	23.0 (3.1)^‡^	23.3 (3.1)^‡^	23.8 (3.2)^‡^	24.1 (3.3)^‡^
Waist circumference, cm	83.3 (8.8)	<0.001	79.9 (9.2)^‡^	79.9 (8.9)^‡^	81.0 (8.8)^‡^	82.1 (8.6)	83.3 (8.7)^‡^	84.2 (8.6)^‡^	85.4 (8.6)^‡^	86.4 (8.7)^‡^
Systolic blood pressure, mmHg	129 (17)	<0.001	124 (19)^†^	123 (17)^‡^	125 (17)^‡^	127 (17)	128 (17)^‡^	130 (17)^‡^	133 (17)^‡^	135 (17)^‡^
Diastolic blood pressure, mmHg	76 (11)	<0.001	73 (11)^‡^	73 (11)^‡^	74 (10)^‡^	75 (11)	76 (10)^‡^	77 (10)^‡^	79 (11)^‡^	79 (11)^‡^
HDL cholesterol, mg/dL	63 (16)	<0.001	64 (16)	66 (16)^‡^	65 (16)^‡^	64 (16)	63 (16)^‡^	62 (16)^‡^	61 (16)^‡^	60 (16)^‡^
LDL cholesterol, mg/dL	127 (30)	<0.001	122 (32)^†^	123 (30)^‡^	125 (29)^‡^	126 (29)	127 (30)^‡^	127 (30)^‡^	128 (30)^‡^	127 (31)^‡^
Triglycerides, mg/dL	113 (70)	<0.001	103 (61)	98 (61)^‡^	102 (61)^‡^	107 (63)	112 (67)^‡^	116 (71)^‡^	123 (79)^‡^	131 (86)^‡^
AST, U/L	24 (9)	<0.001	24 (9)	24 (9)*	23 (8)	23 (8)	24 (9)^†^	24 (9)^‡^	25 (10)^‡^	26 (13)^‡^
ALT, U/L	21 (12)	<0.001	20 (14)	19 (11)^‡^	19 (11)^‡^	20 (11)	21 (12)^‡^	22 (13)^‡^	23 (14)^‡^	25 (16)^‡^
δGTP, U/L	34 (41)	<0.001	29 (39)	28 (31)^‡^	28 (31)^‡^	30 (34)	32 (38)^‡^	36 (42)^‡^	41 (49)^‡^	48 (58)^‡^
Hypertension, %	44.1%		33.8%*	31.8%^‡^	34.7%^‡^	38.4%	42.6%^‡^	46.7%^‡^	53.5%^‡^	60.8%^‡^
Dyslipidaemia, %	54.3%		49.9%	46.9%^‡^	49.3%^‡^	51.9%	54.3%^‡^	56.2%^‡^	58.5%^‡^	60.2%^‡^
Current smoking, %	12.9%		16.5%^‡^	14.1%^‡^	12.3%	12.1%	12.0%	13.2%^‡^	13.9%^‡^	15.2%^‡^
Drinking habit, %										
everyday	21.8%		18.4%	15.5%^‡^	15.8%^‡^	17.4%	20.3%^‡^	23.9%^‡^	28.3%^‡^	33.9%^‡^
sometimes	22.0%		24.5%	21.8%	22.2%	22.1%	21.8%	21.8%	22.5%	21.5%
rarely or never	56.1%		57.1%	62.8%	62.0%	60.5%	57.9%	54.4%	49.2%	44.6%
Walking > 1 hour/day, %	52.8%		46.2%^†^	49.3%^‡^	52.0%	52.1%	52.9%*	53.3%^†^	54%^‡^	54.8%^‡^
**Without cardiovascular disease (n = 171,408)**										
n	171,408		636	8,429	19,066	34,144	38,462	30,405	29,410	10,856
Age, years	63.3 (8.0)	<0.001	62.6 (8.4)	61.9 (8.9)^‡^	62.4 (8.6)^‡^	62.9 (8.3)	63.3 (7.9)^‡^	63.6 (7.7)^‡^	64.0 (7.4)^‡^	64.5 (6.9)^‡^
% Male	38.1%		31.1%	25.3%^‡^	26.8%^‡^	30.9%	36.4%^‡^	42.2%^‡^	49.1%^‡^	56.0%^‡^
BMI, kg/m^2^	23.0 (3.1)	<0.001	21.9 (3.2)^‡^	21.8 (3.0)^‡^	22.1 (3.0)^‡^	22.5 (3.0)	22.9 (3.1)^‡^	23.3 (3.1)^‡^	23.7 (3.2)^‡^	24.1 (3.3)^‡^
Waist circumference, cm	83.2 (8.8)	<0.001	79.6 (9.3)^‡^	79.7 (8.9)^‡^	80.8 (8.8)^‡^	82.0 (8.6)	83.1 (8.7)^‡^	84.1 (8.6)^‡^	85.3 (8.6)^‡^	86.2 (8.7)^‡^
Systolic blood pressure, mmHg	129 (18)	<0.001	124 (19)^†^	123 (17)^‡^	125 (17)^‡^	126 (17)	128 (17)^‡^	130 (17)^‡^	133 (17)^‡^	135 (18)^‡^
Diastolic blood pressure, mmHg	76 (11)	<0.001	73 (11)^‡^	73 (11)^‡^	74 (10)^‡^	75 (11)	76 (11)^‡^	77 (10)^‡^	79 (11)^‡^	80 (11)^‡^
HDL cholesterol, mg/dL	63 (16)	<0.001	64 (17)	66 (16)^‡^	65 (16)^‡^	64 (16)	63 (16)^‡^	62 (16)^‡^	61 (16)^‡^	60 (16)^‡^
LDL cholesterol, mg/dL	127 (30)	<0.001	122 (33)^†^	124 (30)^‡^	125 (30)^‡^	127 (29)	127 (30)^†^	128 (30)^‡^	128 (30)^‡^	127 (31)
Triglycerides, mg/dL	113 (71)	<0.001	102 (61)	98 (61)^‡^	101 (61)^‡^	107 (64)	111 (68)^‡^	117 (72)^‡^	123 (80)^‡^	131 (87)^‡^
AST, U/L	24 (9.2)	<0.001	24 (9.3)	24 (8.6)	23 (8.1)	23 (8.4)	24 (8.8)^†^	24 (9.4)^‡^	25 (9.8)^‡^	26 (13)^‡^
ALT, U/L	21 (12)	<0.001	19 (14)	19 (11)^‡^	19 (10)^‡^	20 (11)	21 (12)^‡^	22 (13)^‡^	23 (14)^‡^	25 (16)^‡^
δGTP, U/L	34 (41)	<0.001	28 (40)	28 (30)^‡^	28 (30)^‡^	30 (34)	32 (37)^‡^	36 (42)^‡^	40 (49)^‡^	47 (58)^‡^
Hypertension, %	42.2%		32.2%*	30.1%^‡^	32.7%^‡^	36.4%	40.7%^‡^	44.9%^‡^	51.7%^‡^	58.7%^‡^
Dyslipidaemia, %	54.0%		49.5%	46.4%^‡^	48.9%^‡^	51.6%	53.8%^‡^	56.1%^‡^	58.1%^‡^	60.1%^‡^
Current smoker, %	13.1%		16.7%^‡^	14.3%^‡^	12.5%	12.3%	12.2%	13.4%^‡^	14.2%^‡^	15.4%^‡^
Drinking habit, %										
everyday	21.7%		18.9%	15.3%^‡^	15.6%^‡^	17.2%	20.2%^‡^	23.6%^‡^	28.1%^‡^	33.9%^‡^
sometimes	22.1%		23.9%	22.2%	22.3%	22.2%	21.9%	21.9%	22.6%	21.4%
rarely or never	56.2%		57.3%	62.5%	62.1%	60.6%	57.9%	54.5%	49.3%	44.7%
Walking > 1 hour/day, %	52.8%		46.1%^†^	49.5%^‡^	52.1%	52.0%	52.9%*	53.4%^†^	54.1%^‡^	54.7%^‡^
**With cardiovascular disease (n = 15,341)**										
n	15,341		54	694	1,652	2,935	3,282	2,673	2,843	1,208
Age, years	66.7 (5.7)	0.058	66.0 (7.0)	66.2 (6.1)	66.2 (6.2)	66.5 (5.8)	66.9 (5.5)	66.8 (5.6)	66.9 (5.5)	66.8 (5.3)
% Male	50.5%		48.1%	35.7%^‡^	37.7%^‡^	44.5%	48.5%^†^	54%^‡^	59.3%^‡^	67.9%^‡^
BMI, kg/m^2^	23.5 (3.1)	<0.001	22.4 (2.6)	22.5 (3.1)^†^	22.7 (3.0)^†^	23.0 (3.0)	23.5 (3.0)^‡^	23.6 (3.0)^‡^	24.0 (3.2)^‡^	24.5 (3.1)^‡^
Waist circumference, cm	84.9 (8.6)	<0.001	83.5 (7.6)	82.0 (8.9)^‡^	82.7 (8.6)^†^	83.7 (8.7)	85.0 (8.4)^‡^	85.3 (8.3)^‡^	86.4 (8.4)^‡^	87.5 (8.3)^‡^
Systolic blood pressure, mmHg	131 (17)	<0.001	126 (16)	127 (16)	128 (16)	129 (16)	131 (16)^‡^	132 (16)^‡^	134 (17)^‡^	136 (17)^‡^
Diastolic blood pressure, mmHg	77 (10)	<0.001	74 (10)	75 (10)*	76 (10)	76 (10)	77 (10)	78 (10)^‡^	78 (10)^‡^	79 (10)^‡^
HDL cholesterol, mg/dL	61 (16)	<0.001	61 (15)	63 (16)	63 (16)	62 (16)	60 (15)*	60 (15)^‡^	59 (15)^‡^	58 (15)^‡^
LDL cholesterol, mg/dL	121 (29)	<0.001	117 (24)	118 (29)	121 (28)	121 (29)	123 (28)	121 (29)	122 (29)	121 (30)
Triglycerides, mg/dL	114 (64)	<0.001	115 (68)	103 (65)	105 (59)	108 (60)	114 (61)^†^	115 (60)^‡^	122 (68)^‡^	128 (75)^‡^
AST, U/L	25 (11)	<0.001	24 (9)	26 (14)	24 (10)	24 (9)	24 (9)	24 (10)	26 (12)^‡^	27 (14)^‡^
ALT, U/L	22 (13)	<0.001	20 (14)	21 (16)	20 (11)	21 (12)	21 (12)	22 (14)*	24 (14)^‡^	25 (16)^‡^
δGTP, U/L	38 (46)	<0.001	34 (26)	32 (42)	31 (35)	34 (41)	36 (42)	39 (49)^‡^	44 (50)^‡^	52 (57)^‡^
Hypertension, %	65.9%		51.9%	52.4%^‡^	57.7%*	60.7%	65.3%^‡^	67.6%^‡^	72.8%^‡^	79.7%^‡^
Dyslipidaemia, %	58.5%		53.7%	53.0%	54.4%	56.1%	59.6%^†^	57.7%	62.6%^‡^	61.6%^†^
Current smoking, %	10.6%		14.8%	11.8%	10.4%	9.4%	10.5%	10.7%	10.7%	13.6%^‡^
Drinking habit, %										
everyday	23.9%		13.0%	17.3%	17.4%	19.0%	21.8%^†^	26.6%^‡^	30.2%^‡^	34.1%^‡^
sometimes	21.1%		31.5%	16.9%	21.2%	21.6%	20.8%	20.7%	21.3%	22.7%
rarely or never	55.0%		55.6%	65.8%	61.4%	59.4%	57.4%	52.7%	48.5%	43.1%
Walking > 1 hour/day, %	52.7%		48.1%	47.7%*	51.6%	52.6%	53.4%	52.0%	53.8%	55.3%

Values are mean (SD) or %. *p < 0.05, ^†^p < 0.01, and ^‡^p < 0.001 vs. 85–89 mg/dL.

The characteristics of the participants without or with a history of CVD are also shown in Table 1. The number of participants without a history of CVD was 171,408 with an average age of 63.3 years; 38.1% were male, and their average BMI was 23.0 kg/m^2^. The participants without a history of CVD showed almost the same characteristics with those of total participants: age, male gender, dyslipidaemia, everyday drinking, and current smoking showed J shape curves. The number of participants with a CVD history was 15,341 with an average age of 66.7 years; 50.5% were males, and the average BMI was 23.5 kg/m^2^. The participants with a history of CVD showed a J shape curve in male gender.

### New-onset diabetes mellitus in total participants

Unadjusted and multivariable-adjusted logistic regression models of total participants in Table 2 show the association between fasting plasma glucose levels and the risk of new-onset diabetes mellitus. In total participants, the unadjusted odds ratio (OR) increased when fasting plasma glucose levels were ≥90 mg/dL. The OR for fasting plasma glucose levels <70 mg/dL elevated after adjustments for sex, age, BMI (model 2) (OR 1.74, 95% CI 1.02–2.98), and remained elevated after adjustments for sex, age, BMI, current smoking status, hypertension, dyslipidaemia and drinking habit (model 5) (OR 1.80, 95% CI 1.05–3.09). The OR for fasting plasma glucose levels ≥90 mg/dL remained increased after the adjustments (model 2–5).

### New-onset diabetes mellitus in participants without or with CVD

**Figure 1:**
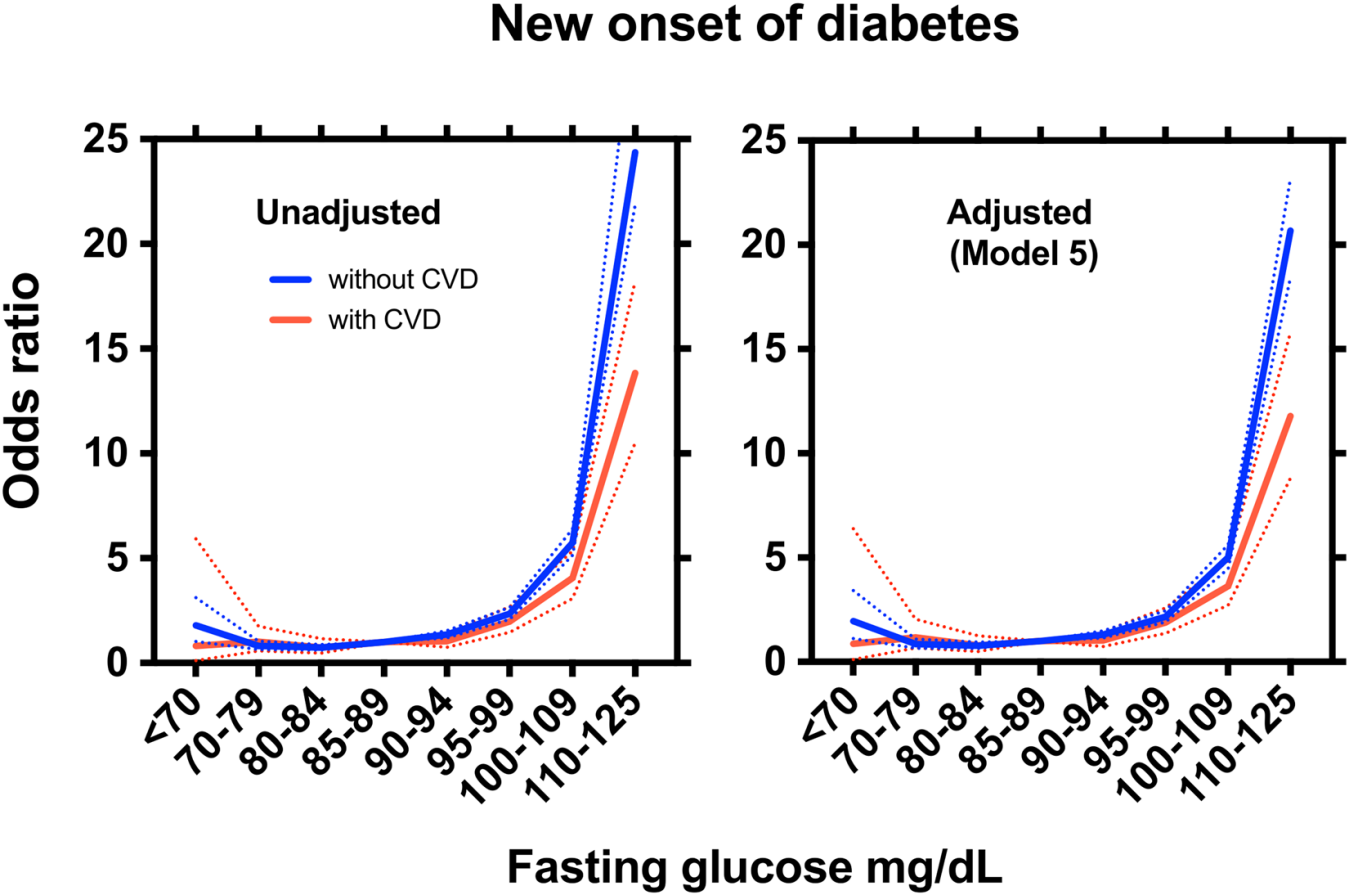
Unadjusted and adjusted odds ratio for new-onset diabetes mellitus. Among participants who underwent Japanese nation-wide annual health check program, The Specific Health Check and Guidance System (SHCG) in 2008, non-diabetic participants without (n = 171,408 blue line) or with (n = 15,341 red line) a history of cardiovascular diseases (CVD), between the age of 40 and 74 years, were selected and divided to 8 categories according to the fasting plasma glucose levels: less than 70 mg/dL, 70 to 79 mg/dL, 80 to 84 mg/dL, 85 to 89 mg/dL, 90 to 94 mg/dL, 95 to 99 mg/dL, 100 to 109 mg/dL, and 110 to 125 mg/dL. The odds ratios (OR) for new-onset diabetes mellitus in the 3-year follow up were calculated with 85 to 89 mg/dL as the reference category. Unadjusted and adjusted (Model 5, adjusted for sex, age, BMI, current smoking, drinking habit, hypertension, dyslipidaemia) odds ratios are shown as solid (ORs) and dotted lines (95% confidential intervals). P values vs the reference category, see Table 2.

In participants without a CVD history, the unadjusted ORs were significantly changed at fasting plasma glucose levels <70 mg/dL or ≥90 mg/dL. The association remained similar after adjustments (model 2–5). The multivariable-adjusted ORs steadily increased for fasting plasma glucose levels ≥90 mg/dL. Meanwhile, in participants with a history of CVD, the unadjusted OR and the multivariable-adjusted ORs for the fasting plasma glucose levels from <70 mg/dL to 90–94 mg/dL were not significantly changed. The unadjusted OR and the multivariable-adjusted ORs steadily increased for fasting plasma glucose levels 95–99 mg/dL (model5) (OR 1.88, 95%CI 1.37–2.59), 100–109 mg/dL (model5) (OR 3.63, 95%CI 2.72–4.85) and 110–125 mg/dL (model5) (OR 11.79, 95%CI 8.80–15.79). The unadjusted (Model 1, left) and multivariable-adjusted (Model 5, right) ORs of new-onset diabetes mellitus for 8 fasting plasma glucose categories in participants without a history of CVD showed a J shape curve (Fig. 1).

### Missing data

Dataset 1 (n = 303,654, all participants) and Dataset 2 (n = 224,784, visited only in 2008 or logical outliers for fasting plasma glucose) showed missing completely at random (Supplementary Fig. 1).

### Propensity score matching

As shown in Supplementary Fig. 3A,B, propensity score densities and distribution were balanced between <70 mg/dL and 85–90 mg/dL groups. Unadjusted (Model 1) and multivariable-adjusted (Model 2–5) logistic regression models showed consistent outcomes in matched (Supplementary Fig. 3) and unmatched datasets (Table 2).

**Table 2:** Unadjusted and multivariable-adjusted ORs (95% CI) for the risk of new-onset diabetes mellitus.

Fasting plasma glucose	Model 1 (Unadjusted)		Model 2		Model 3		Model 4		Model 5	
	OR (95% CI)	p	OR (95% CI)	p	OR (95% CI)	p	OR (95% CI)	p	OR (95% CI)	p
**Total participants (n = 186,749)**										
<**70 mg/dL**	1.65 (0.96–2.81)	0.069	1.74 (1.02–2.98)	0.044	1.72 (1.00–2.94)	0.048	1.73 (1.01–2.96)	0.046	1.80 (1.05–3.09)	0.032
**70–79 mg/dL**	0.84 (0.67–1.04)	0.113	0.91 (0.73–1.14)	0.422	0.91 (0.73–1.13)	0.394	0.92 (0.74–1.15)	0.474	0.90 (0.71–1.13)	0.354
**80–84 mg/dL**	0.74 (0.62–0.87)	<0.001	0.77 (0.65–0.91)	0.003	0.77 (0.65–0.91)	0.002	0.78 (0.65–0.92)	0.003	0.78 (0.71–1.13)	0.006
**85–89 mg/dL**	1.00 (reference)		1.00 (reference)		1.00 (reference)		1.00 (reference)		1.00 (reference)	
**90–94 mg/dL**	1.31 (1.16–1.48)	<0.001	1.25 (1.11–1.41)	<0.001	1.25 (1.11–1.41)	<0.001	1.24 (1.10–1.39)	<0.001	1.27 (1.12–1.43)	<0.001
**95–99 mg/dL**	2.30 (2.06–2.57)	<0.001	2.10 (1.88–2.35)	<0.001	2.10 (1.88–2.35)	<0.001	2.06 (1.83–2.31)	<0.001	2.11 (1.88–2.37)	<0.001
**100–109 mg/dL**	5.51 (4.97–6.10)	<0.001	4.78 (4.32–5.30)	<0.001	4.79 (4.32–5.31)	<0.001	4.64 (4.18–5.14)	<0.001	4.80 (4.31–5.34)	<0.001
**110–125 mg/dL**	22.9 (20.7–25.4)	<0.001	19.2 (17.3–21.3)	<0.001	19.2 (17.3–21.3)	<0.001	18.4 (16.6–20.4)	<0.001	19.3 (17.3–21.4)	<0.001
**Without cardiovascular disease (n = 171,408)**										
<**70 mg/dL**	1.79 (1.02–3.12)	0.041	1.89 (1.08–3.31)	0.026	1.87 (1.07–3.27)	0.028	1.88 (1.08–3.29)	0.027	1.96 (1.12–3.43)	0.019
**70–79 mg/dL**	0.81 (0.64–1.03)	0.090	0.89 (0.69–1.13)	0.326	0.88 (0.69–1.12)	0.301	0.89 (0.70–1.14)	0.359	0.85 (0.66–1.10)	0.213
**80–84 mg/dL**	0.73 (0.61–0.88)	0.001	0.77 (0.64–0.92)	0.005	0.77 (0.64–0.92)	0.005	0.77 (0.64–0.93)	0.006	0.78 (0.65–0.94)	0.010
**85–89 mg/dL**	1.00 (reference)		1.00 (reference)		1.00 (reference)		1.00 (reference)		1.00 (reference)	
**90–94 mg/dL**	1.36 (1.19–1.54)	<0.001	1.29 (1.14–1.47)	<0.001	1.30 (1.14–1.47)	<0.001	1.29 (1.13–1.46)	<0.001	1.31 (1.15–1.50)	<0.001
**95–99 mg/dL**	2.35 (2.08–2.66)	<0.001	2.15 (1.91–2.43)	<0.001	2.16 (1.91–2.43)	<0.001	2.12 (1.87–2.39)	<0.001	2.16 (1.91–2.45)	<0.001
**100–109 mg/dL**	5.73 (5.14–6.40)	<0.001	5.01 (4.48–5.59)	<0.001	5.01 (4.49–5.60)	<0.001	4.86 (4.35–5.43)	<0.001	5.01 (4.47–5.62)	<0.001
**110–125 mg/dL**	24.4 (21.9–27.2)	<0.001	20.6 (18.5–23.0)	<0.001	20.6 (18.5–23.0)	<0.001	19.8 (17.7–22.0)	<0.001	20.7 (18.4–23.1)	<0.001
**With cardiovascular disease (n = 15,341)**										
<**70 mg/dL**	0.81 (0.11–5.93)	0.834	0.84 (0.11–6.14)	0.860	0.82 (0.11–6.03)	0.845	0.82 (0.11–6.08)	0.850	0.86 (0.12–6.38)	0.887
**70–79 mg/dL**	1.01 (0.58–1.75)	0.971	1.08 (0.62–1.88)	0.783	1.07 (0.62–1.86)	0.803	1.10 (0.63–1.92)	0.732	1.17 (0.67–2.05)	0.578
**80–84 mg/dL**	0.74 (0.47–1.15)	0.181	0.78 (0.50–1.21)	0.264	0.77 (0.49–1.21)	0.255	0.78 (0.50–1.21)	0.268	0.79 (0.50–1.26)	0.321
**85–89 mg/dL**	1.00 (reference)		1.00 (reference)		1.00 (reference)		1.00 (reference)		1.00 (reference)	
**90–94 mg/dL**	1.04 (0.75–1.45)	0.807	0.99 (0.71–1.38)	0.963	0.99 (0.71–1.38)	0.947	0.97 (0.70–1.35)	0.860	1.03 (0.73–1.45)	0.870
**95–99 mg/dL**	1.98 (1.46–2.68)	<0.001	1.83 (1.35–2.48)	<0.001	1.83 (1.35–2.48)	<0.001	1.80 (1.33–2.44)	<0.001	1.88 (1.37–2.59)	<0.001
**100–109 mg/dL**	4.05 (3.08–5.34)	<0.001	3.58 (2.72–4.73)	<0.001	3.59 (2.72–4.73)	<0.001	3.45 (2.62–4.56)	<0.001	3.63 (2.72–4.85)	<0.001
**110–125 mg/dL**	13.8 (10.5–18.2)	<0.001	11.7 (8.85–15.5)	<0.001	11.7 (68.8–15.4)	<0.001	11.2 (8.47–14.8)	<0.001	11.8 (8.80–15.8)	<0.001

Model 2 (sex, age, BMI), Model 3 (sex, age, BMI, current smoking), Model 4 (sex, age, BMI, current smoking, hypertension, dyslipidaemia), and Model 5 (sex, age, BMI, current smoking, drinking habit, hypertension, dyslipidaemia). For Model 1 and Model 5 in subjects with or without cardiovascular disease, see also Fig. 1.

## Discussion

The current study presents two major findings regarding the association between normal ranges of fasting plasma glucose and the incidence of diabetes mellitus in a Japanese nation-wide general population. First, when fasting plasma glucose levels were categorized from <70 mg/dL to a maximum of 125 mg/dL in 8 groups, the OR for new-onset diabetes mellitus showed a J-shape curve; the OR increased when fasting plasma glucose levels were <70 mg/dL or ≥90 mg/dL. Second, the OR for new-onset diabetes mellitus increased for the category of fasting plasma glucose levels <70 mg/dL, for participants without a history of CVD.

Previous studies showed that the risk of new-onset diabetes mellitus exhibited a linear increase with fasting plasma glucose levels of 80–125 mg/dL^1–3^ in consistent with our study findings. The OR adjusted for sex, age, and BMI (model 2) was the lowest for fasting plasma glucose levels of 80–84 mg/dL and gradually increased for levels of 85–89 mg/dL, 90–94 mg/dL, 95–99 mg/dL, 100–109 mg/dL, and 110–125 mg/dL. This can be explained by the possibility that an increase in fasting glucose level along with impairment in insulin sensitivity and/or secretion may start 3–6 years before the onset of diabetes mellitus^11^. Our study, the first to investigate the OR for new-onset diabetes mellitus for fasting plasma glucose levels categorized from <70 mg/dL up to 125 mg/dL, showed an increase in OR for fasting plasma glucose levels <70 mg/dL.

The multiple measures nested within each individual could affect the outcomes. Since glucose levels may show year-to-year variations, a multilevel approach should have been considered with time as fixed-effect term in the model. Since only once fasting plasma glucose levels in 2008 were used for our categorization, we assessed the year-to-year reproducibility of measurement. Seventy eight of 502 participants (16%), whose fasting plasma glucose levels were <70 mg/dL in 2008 and measured in 2009, recorded <70 mg/dL again (Supplementary Table 1). Fasting plasma glucose levels <70 mg/dL were reproducible at least in part of the group. In the rest 84% participants, fasting plasma glucose levels were not <70 mg/dL. We considered 3 possibilities: (1) the variability of glucose level was large, so fasting plasma glucose was not <70 mg/dL by chance, (2) impaired glucose tolerance in 2009 and (3) measurement error in 2008. To discuss the variability of glucose levels: (1), we calculated standard deviation (SD) of fasting plasma glucose levels in 2009–2011 whose data was available from 2009 to 2011 (Supplementary Table 2). The SD was high in the group of fasting plasma glucose levels <70 mg/dL in 2008. We need to consider the possibility that the reason of the OR of the new onset of diabetes mellitus increased when fasting plasma glucose levels <70 mg/dL is because of the variability of fasting plasma glucose level. To obtain support to our conclusion, we calculated ORs for the risk of new-onset diabetes mellitus in participants < 70mg/dL at least once during 2008–2011 as compared to reference controls who continued to show 85–89 mg/dL during three continuous visits (2008–2011) (Supplementary Table 3). Results support our notion that ORs were increased in participants < 70mg/dL as compared to 85–89 mg/dL.

Characteristics of the group with fasting plasma glucose levels < 70mg/dL was previously reported^6^. In a total of 1,197,384 Korean general population^6^, ones with fasting glucose levels <70 mg/dL showed a high percentage of current smoking and low physical activity, which were observed also in our participants with the same category.

Current smoking has been reported as a risk factor for the onset of diabetes mellitus^12,13^. We thus compared new onset of diabetes mellitus between non-current and current smoking. In total participants, the incident of diabetes mellitus was 3.7% in non-current smoking vs 4.9% in current smoking (p < 0.001, χ^2^). As shown in Supplementary Table 4, the incidence of diabetes mellitus was higher in current smoking than in non-current smoking among ones without CVD (4.7% vs 3.5%, p < 0.001), and the incidence tends to be increased in the subgroup with fasting plasma glucose <70 mg/dL (3.8% vs 1.7%, p = 0.17). The mechanism(s) how current smoking increases the risk for diabetes mellitus has not been clarified^12,13^. Wu *et al*. proposed a possible hypothesis: nicotine, a major constitute of cigarette smoke, increases lipolysis, which causes body weight reduction, elevates the levels of circulating free fatty acids and thus causes insulin resistance in insulin sensitive tissues^14^. Elevation of circulating free fatty acids, induced by smoking-induced lipolysis, could simultaneously cause hyperinsulinemia and thereby induce reactive hypoglycaemia^15–17^. Taken together, new-onset diabetes mellitus in current smoking with levels <70 mg/dL could be caused by insulin resistance and hyperinsulinemic hypoglycaemia, although those were not assessed in the current study. Since fasting plasma glucose <70 mg/dL remained an independent risk factor for new-onset diabetes mellitus after adjustments for current smoking and its related factors, sex, age, BMI, hypertension, dyslipidaemia and drinking habit (model 3–5), other unknown mechanism(s) is needed to be considered.

It is well known that low physical activity is the risk for the onset of diabetes mellitus^18–20^. Our participants with fasting plasma glucose <70 and 70–79 mg/dL showed low frequency in walking >1 hour/day, suggesting low physical activity. Since the participants with <70 mg/dL also showed low BMI, low waist circumference and low LDL-C (Table 1), this category may include a group with nutritional deficiencies and/or low muscle mass and functionality, which can be comorbid with low physical activity^21^. Like obesity and ageing, loss of muscle mass and functionality, termed sarcopenia, can be a key driver of type 2 diabetes^21^. Future study is needed to clarify this point in populations of this category.

Wei *et al*. reported that the relation between fasting plasma glucose and the risk for CVD showed a J shape curve: the onset of CVD increased in those with fasting plasma glucose levels <70mg/dL compared to that in those with levels of 85–109 mg/dL^4^. We therefore assessed the ORs for new-onset diabetes mellitus separately in participants with or without a history of CVD. The OR of the group of fasting plasma glucose level <70 mg/dL increased in those without a history of CVD, but not in those with a history of CVD. We could not obtain the reason why the OR in <70 mg/dL was only increased in participants without a history of CVD. Possible reasons may be that age (66.7 vs 63.3 years, p < 0.001), male gender (50.5% vs 38.1%, p < 0.001), BMI (23.5 vs 23.0 kg/m^2^, p < 0.001) and waist circumference (84.9 vs 83.2 cm, p < 0.001) were higher in participants with CVD as compared in those without CVD. In any case, new onset diabetes in participants without a history of CVD can be a risk for future onset of CVD. The possible mechanisms for the link between the onset of CVD and fasting plasma glucose levels < 70 mg/dL have been proposed: (1) long-term exposure to low fasting plasma glucose may serve as a risk factor for CVD, possibly through abnormal cardiac activity and thrombosis, particularly in patients with atherosclerosis;^4,7^ (2) hypoglycaemia or rapid changes in plasma glucose may lead to elevations of counter-regulatory hormones^8^. Current study provides the notion that an increase in new onset diabetes may also increase the risk of CVD in the category of plasma glucose levels <70mg/dL.

Our study has several limitations. First, we evaluated only fasting plasma glucose level. Neither oral glucose tolerance test data, nor postprandial plasma glucose level measurements of the participants were available to us; hence we could not consider post-challenge or postprandial glucose levels, which are better markers for the onset of diabetes mellitus. Second, conditions of blood sampling were self-reported so there was a possibility of postprandial plasma glucose level were in the data. Also, we could not get information about medication. The use of drugs affecting glucose intolerance may bias the current analysis. Third, history of CVD and drug medication use was based on data from a self-administered questionnaire. When the participants were uncertain about the response, medical staff provided assistance; hence, the responses were considered accurate. Fourth, insulin resistance could not be assessed in our study, because we did not measure insulin levels. Fifth, since only 38.9% of targets in all Japan had undergone SHCG in 2008 and the available data were limited to 26 among 47 prefectures in Japan, data might have sampling bias. Sixth, our participants consisted of a 40–74 years-old Japanese population; hence, our findings cannot be generalized to other race/ethnic groups. Seventh, there was no information on smoking dose, exposure amount and smoking history in the study. Future analysis may be required to evaluate relationship between smoking status and exposure and onset of diabetes mellitus.

In conclusion, the risk for new-onset diabetes mellitus was high when fasting glucose levels were <70 mg/dL in participants without history of CVD, indicating that this range of fasting glucose is a new and significant risk factor for diabetes mellitus within the general population. Considering that this range is also a risk factor for CVD, it is important to elucidate the relation between CVD and diabetes mellitus in cases of low fasting glucose levels (<70 mg/dL).

## Methods

### Study population

This study used data of the annual health check program, “The Specific Health Check and Guidance System” (SHCG) in Japan^22–25^, launched by the Ministry of Health, Labour and Welfare (MHLW), Japan in 2008. The target of SHCG was the Japanese general population between the ages of 40 and 74 years, estimated to be 51,919,920. The current study was performed as a part of the ongoing project “Design of the comprehensive healthcare system for chronic kidney disease (CKD) based on the individual risk assessment by Specific Health Check-ups.” We asked municipalities through the whole country to sign a contract to provide data, and we were able to contract with 199 municipalities belonging to 26 prefectures (Hokkaido, Miyagi, Yamagata, Ibaraki, Tochigi, Tokyo, Saitama, Chiba, Kanagawa, Niigata, Nagano, Ishikawa, Fukui, Gifu, Osaka, Hyogo, Okayama, Tokushima, Kochi, Fukuoka, Saga, Nagasaki, Oita, Kumamoto, Miyazaki, and Okinawa) all over Japan. This database is the largest nation-wide scale samples with personal data matched over the years. The individual data of the SHCG from 2008 to 2011 had been sent to and verified by an independent data centre, the NPO Japan Clinical Support Unit (Tokyo, Japan)^23,25^.

In the participants from 26 prefectures, we excluded ones who visited only once in 2008 (n = 78,509) and ones with incomplete information recorded in the database, such as data about sex, age, BMI, waist circumference, SBP, DBP, fasting plasma glucose levels, high-density lipoprotein-cholesterol (HDL-C) levels, low-density lipoprotein-cholesterol (LDL-C) levels, triglyceride (TG) levels, smoking habits, history of heart disease, and history of stroke (n = 38,396) (Supplementary Fig. 1). We finally selected 186,749 participants without diabetes mellitus (see definition below) in 2008.

### Ethics approval and consent to participate

All procedures performed in studies involving human participants were in accordance with the ethical standards of the institutional and/or national research committee at which the studies were conducted (Fukushima Medical University; IRB Approval Number #1485, #2771) and with the 1964 Helsinki declaration and its later amendments or comparable ethical standards. This study was conducted according also to the Ethical Guidelines for Medical and Health Research Involving Human Subjects enacted by MHLW of Japan [http://www.mhlw.go.jp/file/06-Seisakujouhou-10600000-Daijinkanboukouseikagakuka/0000069410.pdf and http://www.mhlw.go.jp/file/06-Seisakujouhou-10600000-Daijinkanboukouseikagakuka/ 0000080278.pdf]. In the context of the guideline, the investigators shall not necessarily be required to obtain informed consent, but we made public information concerning this study on the web [http://www.fmu.ac.jp/univ/sangaku/data/koukai_2/2771.pdf] and ensured the opportunities for the research subjects to refuse utilizing their personal information.

### Measurements

Trained staff measured height, body weight, blood pressure, and waist circumference of each subject. Questionnaires recording data on smoking status (current smoker or not), drinking habits (everyday, sometimes, rarely or never), regular exercise (walking >1 h/day, rarely or never), anti-hypertensive drug use, anti-hyperglycaemic drug use, lipid-lowering drug use, history of heart disease, and history of stroke were administered. Blood samples were collected after an overnight fast and were assayed within 24 hours with automatic clinical chemical analysers. When needed, HbA_1c_ was corrected as a National Glycohemoglobin Standardization Program equivalent value, calculated with the following formula: HbA_1c_ (%) = HbA_1c_ (Japan Diabetes Society) (%) +0.4%^26^.

### Definition of diabetes mellitus, hypertension, dyslipidaemia, and history of CVD

For this study, a participant was considered to have diabetes mellitus when the fasting plasma glucose level was ≥126 mg/dL, when HbA_1c_ levels were ≥6.5% (48 mmol/mol), or if the participant had self-reported the use of anti-hyperglycaemic drugs in 2008. Participants were considered to have new-onset diabetes mellitus if they met the above diabetes criteria in 2009, 2010, or 2011. Participants were considered hypertension if their SBP was ≥140 mmHg, if their DBP was ≥90 mmHg, or if they had self-reported the use of antihypertensive drugs. Participants were considered dyslipidaemia if HDL-C levels were <40 mg/dL (1.0 mmol/L), if LDL-C levels were ≥140 mg/dL (3.6 mmol/L), if TG levels were ≥150 mg/dL (1.7 mmol/L), or if they had self-reported the use of lipid lowering drugs. Participants were considered to have a history of CVD if they had a self-reported history of coronary heart disease (angina pectoris and myocardial infarction) or stroke in 2008. Other atherosclerotic cardiovascular disease such as peripheral arterial disease, aortic aneurism and carotid artery disease were not included in this study.

### Statistical analyses

To study the association between new-onset diabetes mellitus and low fasting plasma glucose levels, we analysed 8 categories of fasting plasma glucose levels: <70mg/dL, 70–79 mg/dL, 80–84 mg/dL, 85–89 mg/dL, 90–94 mg/dL, 95–99 mg/dL, 100–109 mg/dL, and 110–125 mg/dL. The group with fasting plasma glucose level of 85–89 mg/dL was used as the reference category. Considering the previous study for the reference (85–99 mg/dL)^6^, we chose a narrower range (5 mg/dL vs 15 mg/dL) to analyse more precisely the impact of fasting glycaemic ranges. One-way analysis of variance (ANOVA) followed by Dunnett’s post-hoc test or χ^2^ was used to compare group means. Unadjusted and multivariable-adjusted logistic regression models were used to estimate the association between fasting plasma glucose concentration and the risk of new-onset diabetes mellitus in 1–3 years follow-ups. In the first step, we carried out unadjusted analyses (Model 1). In the second step, we adjusted for age, sex, and BMI (Model 2). In the third step, we further adjusted for current smoking (Model 3). In the fourth step, we further adjusted for hypertension and dyslipidaemia (Model 4). In the last step, we finally adjusted for drinking habits (Model 5). In order to validate assumptions of multivariate models, multicollinearity of cofactors was evaluated by scatter plot analysis and outliners were assessed for residual errors. Main analysis was performed on the list-wise deletion dataset (complete case, see below), and consistency of the results were assessed by multiple imputation and propensity score matched dataset as bellow. Analyses were performed using SPSS software (version 24.0; SPSS, Chicago, IL, USA) or R 3.4.3.

#### Missing data

Among all participants (n = 303,654) in 2008 (Dataset1), missing data were compared between ones who visited only in 2008 (n = 78,334) or showed logical outliers for fasting plasma glucose (n = 536) (Dataset 2) and the other (Supplementary Fig. 2)^27^. The main analysis were done on the complete case (Dataset 3) excluded for missing information (n = 38,035): sex, age, BMI, waist circumference, systolic and diastolic blood pressure, fasting plasma glucose, HbA1c, HDL- and LDL-cholesterol, triglycerides, smoking habits, history of heart disease, and history of stroke.

#### Propensity score matching

Propensity scores were calculated using logistic models of potential covariates (age, sex, body weight, waist circumference, systolic and diastolic blood pressure, triglycerides, LDL- and HDL-cholesterol, and presence of hypertension and dyslipidaemia) in participants with fasting plasma glucose <70 mg/dL or 85–90 mg/dL in 2008, and pairs matched for propensity scores were selected by 1:1 (Supplementary Fig. 3)^28^. After matching distances were estimated by logit regression, nearest neighbor and non-caliper sampling without replacement was used. Distribution before and after propensity score matching were assessed by descriptive statistics values and standardized mean differences. Unadjusted (Model 1) and multivariable-adjusted (Model 2–5) logistic regression models were also calculated by using the propensity score matched groups (<70 mg/dL vs 85–90 mg/dL) to estimate the risk of new-onset diabetes mellitus.

## Electronic supplementary material

Supplement Figure 1-3, Table 1-4

## References

[1] Tirosh A (2005). Normal fasting plasma glucose levels and type 2 diabetes in young men. The New England journal of medicine.

[2] Nichols GA, Hillier TA, Brown JB (2008). Normal fasting plasma glucose and risk of type 2 diabetes diagnosis. The American journal of medicine.

[3] Brambilla P (2011). Normal fasting plasma glucose and risk of type 2 diabetes. Diabetes care.

[4] Wei M (2000). Low fasting plasma glucose level as a predictor of cardiovascular disease and all-cause mortality. Circulation.

[5] Sarwar N (2010). Diabetes mellitus, fasting blood glucose concentration, and risk of vascular disease: a collaborative meta-analysis of 102 prospective studies. Lancet (London, England).

[6] Park C (2013). Fasting glucose level and the risk of incident atherosclerotic cardiovascular diseases. Diabetes care.

[7] Hanefeld M, Duetting E, Bramlage P (2013). Cardiac implications of hypoglycaemia in patients with diabetes - a systematic review. Cardiovascular diabetology.

[8] Tanne D, Koren-Morag N, Goldbourt U (2004). Fasting plasma glucose and risk of incident ischemic stroke or transient ischemic attacks: a prospective cohort study. Stroke.

[9] Cryer PE (2009). Evaluation and management of adult hypoglycemic disorders: an Endocrine Society Clinical Practice Guideline. The Journal of clinical endocrinology and metabolism.

[10] Conn JW, Fajans SS (1956). & Seltzer, H. S. Spontaneous hypoglycemia as an early manifestation of diabetes mellitus. Diabetes.

[11] Tabak AG (2009). Trajectories of glycaemia, insulin sensitivity, and insulin secretion before diagnosis of type 2 diabetes: an analysis from the Whitehall II study. Lancet (London, England).

[12] Willi C, Bodenmann P, Ghali WA, Faris PD, Cornuz J (2007). Active smoking and the risk of type 2 diabetes: a systematic review and meta-analysis. Jama.

[13] Pan A, Wang Y, Talaei M, Hu FB, Wu T (2015). Relation of active, passive, and quitting smoking with incident type 2 diabetes: a systematic review and meta-analysis. *The lancet. Diabetes &*. endocrinology.

[14] Wu Y (2015). Activation of AMPKalpha2 in adipocytes is essential for nicotine-induced insulin resistance *in vivo*. Nature medicine.

[15] Unger RH (1995). Lipotoxicity in the pathogenesis of obesity-dependent NIDDM. Genetic and clinical implications. Diabetes.

[16] Shimabukuro M (1997). Direct antidiabetic effect of leptin through triglyceride depletion of tissues. Proceedings of the National Academy of Sciences of the United States of America.

[17] McGarry JD, Dobbins RL (1999). Fatty acids, lipotoxicity and insulin secretion. Diabetologia.

[18] Ekelund U (2012). Physical activity reduces the risk of incident type 2 diabetes in general and in abdominally lean and obese men and women: the EPIC-InterAct Study. Diabetologia.

[19] Aune D, Norat T, Leitzmann M, Tonstad S, Vatten LJ (2015). Physical activity and the risk of type 2 diabetes: a systematic review and dose-response meta-analysis. European journal of epidemiology.

[20] Cloostermans L (2015). Independent and combined effects of physical activity and body mass index on the development of Type 2 Diabetes - a meta-analysis of 9 prospective cohort studies. The international journal of behavioral nutrition and physical activity.

[21] Roden M (2015). Future of muscle research in diabetes: a look into the crystal ball. Diabetologia.

[22] Iseki K (2012). Risk factor profiles based on estimated glomerular filtration rate and dipstick proteinuria among participants of the Specific Health Check and Guidance System in Japan 2008. Clinical and experimental nephrology.

[23] Wakasugi M (2014). Association between combined lifestyle factors and non-restorative sleep in Japan: a cross-sectional study based on a Japanese health database. PloS one.

[24] Yano Y (2015). Long-Term Blood Pressure Variability, New-Onset Diabetes Mellitus, and New-Onset Chronic Kidney Disease in the Japanese General Population. Hypertension (Dallas, Tex.: 1979).

[25] Hasegawa K (2016). Control Status of Atherosclerotic Cardiovascular Risk Factors Among Japanese High-Risk Subjects:Analyses of a Japanese Health Check Database from 2008 to 2011. Journal of atherosclerosis and thrombosis.

[26] Seino Y (2010). Report of the committee on the classification and diagnostic criteria of diabetes mellitus. Journal of diabetes investigation.

[27] Schafer JL, Olsen MK (1998). Multiple Imputation for Multivariate Missing-Data Problems: A Data Analyst’s Perspective. Multivariate behavioral research.

[28] Ho DE, Imai K, King G, Stuart EA (2007). Matching as Nonparametric Preprocessing for Reducing Model Dependence in Parametric Causal Inference. Political Analysis.

