# Supplementary material for: Low fasting plasma glucose level as a predictor of new-onset diabetes mellitus on a large cohort from a Japanese general population
Source: Sci Rep. 2018 Sep 17;8:13927. doi: 10.1038/s41598-018-31744-4 (PMC6141503; doi:10.1038/s41598-018-31744-4)
Supplement: Supplementary file 1 — Supplement Figure 1-3, Table 1-4 [file 41598_2018_31744_MOESM1_ESM.zip › ST4.pdf]

Supplementary table 4. Incidence of new-onset diabetes mellitus in participants with non-current or current smoking

	Without cardiovascular disease (n=171,408)			With cardiovascular disease (n=15,341)		
Fasting plasma glucose	Non-current smoking	Current smoking	p	Non-current smoking	Current smoking	p
All	3.5%	4.7%	<0.001	5.3%	7.7%	<0.001
<70 mg/dL	1.7%	3.8%	0.17	2.2%	0.0%	0.67
70-79 mg/dL	0.9%	1.4%	0.06	2.5%	1.2%	0.49
80-84 mg/dL	0.8%	1.3%	<0.05	1.7%	1.7%	0.96
85-89 mg/dL	1.1%	1.3%	0.24	2.2%	3.3%	0.25
90-94 mg/dL	1.5%	2.0%	<0.01	2.1%	5.0%	<0.001
95-99 mg/dL	2.6%	2.9%	0.40	4.3%	5.3%	0.46
100-109 mg/dL	6.1%	7.4%	<0.01	8.3%	11.3%	<0.05
110-125 mg/dL	21.6%	25.3%	<0.001	24.0%	26.8%	0.44
