# Supplementary material for: Low fasting plasma glucose level as a predictor of new-onset diabetes mellitus on a large cohort from a Japanese general population
Source: Sci Rep. 2018 Sep 17;8:13927. doi: 10.1038/s41598-018-31744-4 (PMC6141503; doi:10.1038/s41598-018-31744-4)
Supplement: Supplementary file 1 — Supplement Figure 1-3, Table 1-4 [file 41598_2018_31744_MOESM1_ESM.zip › ST1.pdf]

**Supplementary table 1. Fasting plasma glucose during 2009-2011 in participants with < 70m in 20[illegible]**

| year | n | Fasting glucose mg/dL | | Number of fasting plasma glucose < 70mg/dL |
|---|---|---|---|---|
| | | mean | SD | |
| 2009 | 502 | 79.6 | (11.2) | 78 (16%) |
| 2010 | 354 | 81.2 | (11.5) | 36 (10%) |
| 2011 | 104 | 85.3 | (15.3) | 7 (7%) |
