# Supplementary material for: Low fasting plasma glucose level as a predictor of new-onset diabetes mellitus on a large cohort from a Japanese general population
Source: Sci Rep. 2018 Sep 17;8:13927. doi: 10.1038/s41598-018-31744-4 (PMC6141503; doi:10.1038/s41598-018-31744-4)
Supplement: Supplementary file 1 — Supplement Figure 1-3, Table 1-4 [file 41598_2018_31744_MOESM1_ESM.zip › ST2.pdf]

Supplementary table 2. Fasting plasma glucose levels during 2009 to 2011

Fasting plasma glucose in 2008	n	2009		2010		2011	
		mean	SD	mean	SD	mean	SD
All	38781	94.7	(10.0)	94.8	(10.5)	94.7	(10.7)
<70 mg/dL	69	82.6	(11.2)	84.5	(16.4)	85.2	(11.0)
70-79 mg/dL	1363	82.9	(7.2)	83.2	(7.2)	83.6	(8.3)
80-84 mg/dL	3720	86.2	(6.4)	86.5	(7.0)	86.3	(6.8)
85-89 mg/dL	7094	89.2	(6.1)	89.4	(6.9)	89.4	(7.1)
90-94 mg/dL	8903	92.7	(6.6)	92.8	(7.9)	92.6	(7.0)
95-99 mg/dL	7443	96.2	(7.1)	96.2	(7.4)	96.1	(7.7)
100-109 mg/dL	7528	101.5	(8.4)	101.5	(9.0)	101.5	(9.4)
110-125 mg/dL	2661	110.5	(11.2)	110.5	(12.4)	111.0	(14.1)
