# Supplementary material for: Low fasting plasma glucose level as a predictor of new-onset diabetes mellitus on a large cohort from a Japanese general population
Source: Sci Rep. 2018 Sep 17;8:13927. doi: 10.1038/s41598-018-31744-4 (PMC6141503; doi:10.1038/s41598-018-31744-4)
Supplement: Supplementary file 1 — Supplement Figure 1-3, Table 1-4 [file 41598_2018_31744_MOESM1_ESM.zip › ST3.pdf]

Supplementary Tab3e 4. Unadjusted and multivariable-adjusted ORs (95% CI) for the risk of new-onset diabetes mellitus in participants <70mg/dL at least once during 2008-2011 as compared to reference controls who continued to show 85-90 mg/dL during 2008-2011

	Model 1 (Unadjusted)		Model 2		Model 3		Model 4		Model 5	
Fasting plasma glucose	OR (95% CI)	p	OR (95% CI)	p	OR (95% CI)	p	OR (95% CI)	p	OR (95% CI)	p
<70 mg/dL (n=1590)	3.48 (1.25-9.21)	0.017	3.44 (1.23-9.62)	0.019	2.96 (1.05-8.32)	0.04	2.89 (1.02-8.15)	0.045	2.50 (0.88-7.07)	0.085
85-89 mg/dL (n=461)	1.00 (reference)		1.00 (reference)		1.00 (reference)		1.00 (reference)		1.00 (reference)	

Model 2 (sex, age, BMI), Model 3 (sex, age, BMI, current smoking), Model 4 (sex, age, BMI, current smoking, hypertension, dyslipidemia), and Model 5 (sex, age, BMI, current smoking, drinking habit, hypertension, dyslipidemia).
