# Supplementary figures and images for: Low fasting plasma glucose level as a predictor of new-onset diabetes mellitus on a large cohort from a Japanese general population
Source: Sci Rep. 2018 Sep 17;8:13927. doi: 10.1038/s41598-018-31744-4 (PMC6141503; doi:10.1038/s41598-018-31744-4)

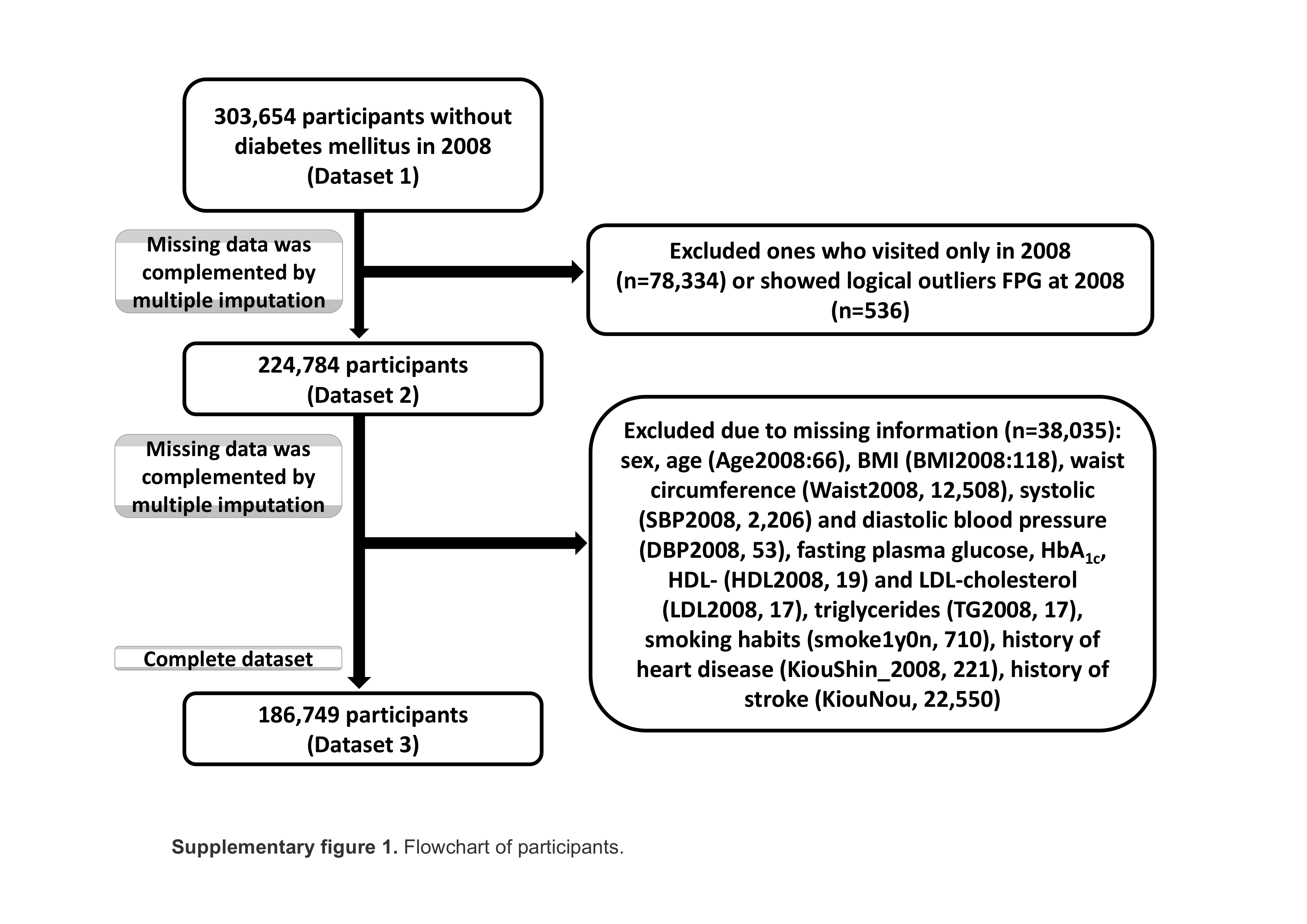

Supplement: Supplementary file 1 — Supplement Figure 1-3, Table 1-4 [file 41598_2018_31744_MOESM1_ESM.zip › S1.tif]

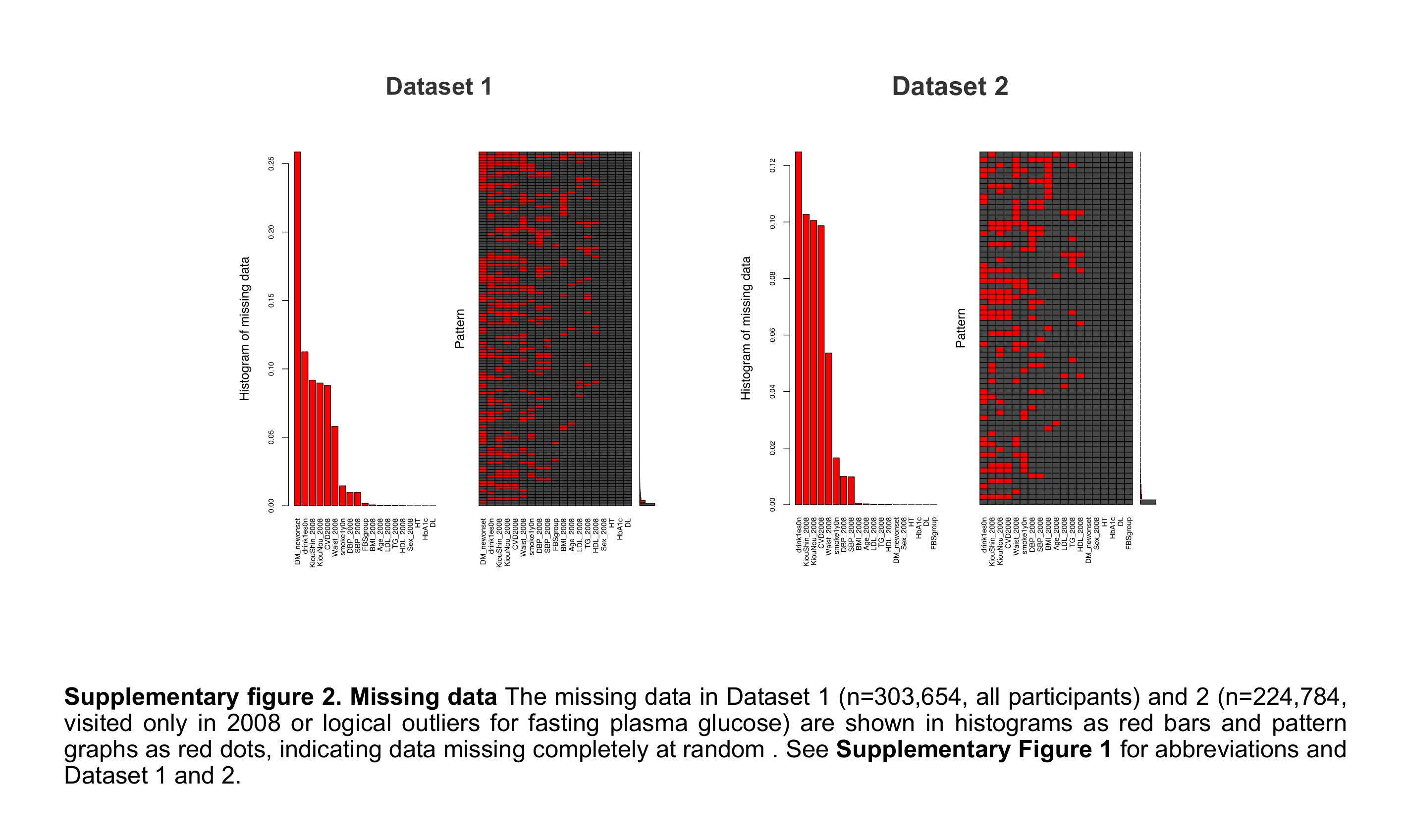

Supplement: Supplementary file 1 — Supplement Figure 1-3, Table 1-4 [file 41598_2018_31744_MOESM1_ESM.zip › S2.tif]

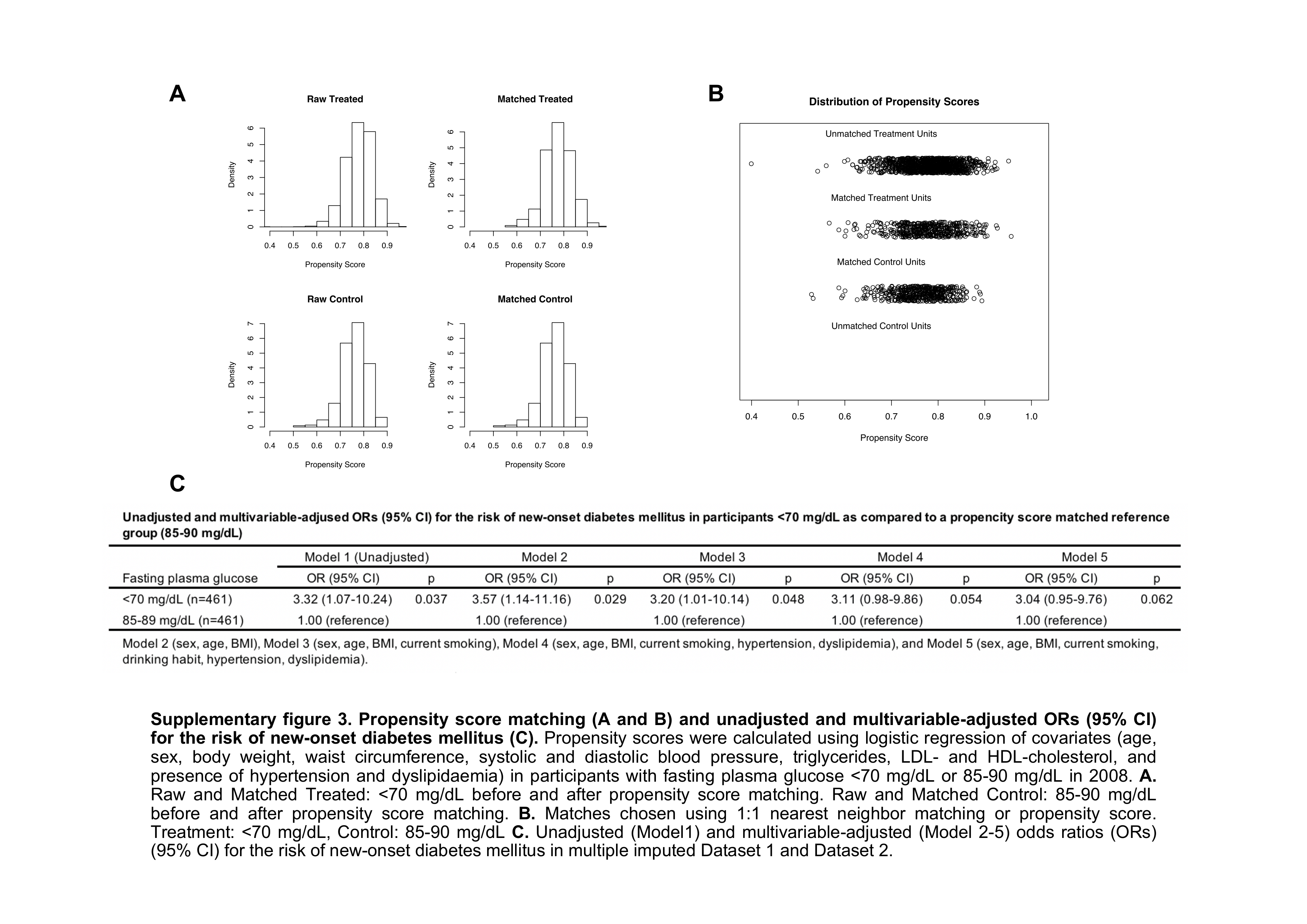

Supplement: Supplementary file 1 — Supplement Figure 1-3, Table 1-4 [file 41598_2018_31744_MOESM1_ESM.zip › S3.tif]
